# Placental transfer and mammary excretion of a novel angiotensin receptor blocker fimasartan in rats

**DOI:** 10.1186/s40360-016-0080-y

**Published:** 2016-07-26

**Authors:** Tae Hwan Kim, Min Gi Kim, Soyoung Shin, Yong-Ha Chi, Soo-Heui Paik, Joo-Han Lee, Sun Dong Yoo, Yu Seok Youn, Jürgen B. Bulitta, Sang Hoon Joo, Seok Won Jeong, Kwon-Yeon Weon, Beom Soo Shin

**Affiliations:** 1 School of Pharmacy, Sungkyunkwan University, Suwon, Gyeonggi-do South Korea; 2 College of Pharmacy, Wonkwang University, Iksan, Jeonbuk South Korea; 3 Boryung Pharm. Co., Ltd., Seoul, South Korea; 4 College of Pharmacy, Sunchon National University, Sunchon, Jeonnam South Korea; 5 College of Pharmacy, University of Florida, Orlando, FL USA; 6 College of Pharmacy, Catholic University of Daegu, 13-13 Hayang-ro, Hayang-eup Gyeongsan-si, Gyeongbuk 712-702 South Korea

**Keywords:** Maternal-fetal transfer, Milk secretion, Hypertension, Angiotensin receptor blocker, Fimasartan

## Abstract

**Background:**

Fimasartan (FMS) is a potent angiotensin receptor blocker for the treatment of mild to moderate hypertension. This study aimed to evaluate the transfer of FMS to fetus and breast milk in rats.

**Methods:**

In order to study the transfer to the fetus and nursing pup, pregnant and nursing maternal rats were administered with FMS by a constant intravenous infusion to reach target plasma concentrations of 200 ng/mL and 100 ng/mL. The concentrations of FMS in plasma, placenta, amniotic fluid, fetus, and milk were determined by a validated LC-MS/MS assay.

**Results:**

Upon constant intravenous infusion, the plasma FMS concentration reached the target steady state concentrations (C_ss_ = 200 ng/mL and 100 ng/mL) in 24 h. The tissue-to-plasma partition coefficients (K_p_) for placenta, amniotic fluid, and milk were obtained based on the observed FMS concentrations in the tissues and C_ss_. The K_p_ values for all tissues were not different between high (C_ss_ = 200 ng/mL) and low (C_ss_ = 100 ng/mL) dose groups. While the mean K_p_ of the placenta was 44.6–59.0 %, the mean K_p_ was 1.3–1.7 % for the amniotic fluid and 14.9–17.0 % for fetus. The mean K_p_ of milk was 10.4–15.2 %.

**Conclusions:**

Placental transfer and milk excretion of FMS was relatively lower compared to other angiotensin receptor blockers.

## Background

Hypertension is one of the most common medical problems encountered during pregnancy, affecting 6–8 % of pregnant women [1]. Although many pregnant women with hypertension have healthy babies without serious problems, high blood pressure can be associated with increased maternal and perinatal risks [1, 2]. For example, the mother develops preeclampsia which may affect placenta as well as the kidney, liver, and brain of the mother and cause fetal complications such as low birth weight and early delivery [1, 2]. In the most serious cases, preeclampsia can progress to a convulsive and life-threatening eclampsia [2].

Nevertheless, treatment of a pregnant woman with a drug may cause various developmental toxicities. Some medications to lower blood pressure are considered safe during pregnancy. However, some of the most effective antihypertensive agents available, such as angiotensin-converting enzyme (ACE) inhibitors and angiotensin receptor blockers (ARBs) are generally avoided during pregnancy [3–7]. For example, losartan (Cozaar®) has been assigned to pregnancy category D by the FDA [8], which implies that there is positive evidence of human fetal risk based on adverse reaction data from investigational or marketing experience or studies in humans.

The fetal toxicities associated with ACE inhibitors and ARBs are likely related to the reduction of angiotensin II and dysfunction of the renin-angiotensin system during fetal development [7]. ACE inhibitors are known to induce fetal toxicity including severe renal dysfunction, neonatal anuria, skull ossification defects, reduced fetal growth, still birth, and dysmorphic features in animals as well as humans [7, 9]. Similarly, intrauterine exposure to ARBs has shown to decrease fetal body weight, induce renal dysfunction, and even lead to fetal death [10]. Evidences of the fetal and/or neonatal toxicity, primarily renal dysfunction associated with the use of ARBs during human pregnancy are available [7, 11, 12].

With regard to lactation, the data on the use of these antihypertensive agents are very limited. The American Academy of Pediatrics (2001) considered only two commonly used ACE inhibitors, captopril and enalapril compatible with breast-feeding. However, there are generally insufficient data on other ACE inhibitors or ARBs to make any recommendations regarding the use and safety during lactation.

Fimasartan (FMS) is a potent angiotensin AT1-selective ARB that has been approved by Korean Ministry of Food and Drug Safety (MFDS) for the treatment of mild to moderate hypertension in 2010 and it has been licensed out to 13 Latin American countries as well as Russia and China. In rats, FMS was rapidly and extensively absorbed with an oral bioavailability of 32.7–49.6 % [13]. In healthy volunteers, FMS absorption was rapid and the exposure was dose proportional with the terminal half-life ranging from 5 to 16 h following oral administration [14, 15]. FMS also showed a good safety profile after a single oral doses of 20 to 480 mg and repeated oral doses of 120 and 360 mg in humans [14]. In patients with arterial hypertension, minimal side effects, evident efficacy, and high overall patient compliance were observed [16].

In addition to the inherent toxic effects of a drug, drug distribution characteristics are directly related to the fetal and neonatal safety. Substantial distribution of a drug into fetus and breast milk is one of the most important factors contributing the fetal and neonatal toxicities while less distribution would lead to less toxicities. Placental transfer and milk excretion of some of the marketed ARBs are known to be significant [17–21]. Distribution of FMS into fetus or breast milk has not been reported, which is critical to evaluate its safety in pregnancy and lactation.

In the present study, the placental transfer and milk excretion of FMS were examined in pregnant and lactating rats. The pharmacokinetic disposition of FMS in the placenta, amniotic fluid, fetus, and milk were evaluated at steady state conditions following intravenous infusion.

## Methods

### Chemicals and reagents

Fimasartan and BR-A-563 (internal standard) were supplied by Boryung Pharm. Co., Ltd. (Seoul, Korea). Zoletil 50 (tiletamine/zolazepam = 125/125 mg) was purchased from Virbac Laboratory (Carros cedex, France). HPLC grade acetonitrile, methanol, and distilled water were purchased from Mallinckrodt Baker, Inc. (Phillipsburg, NJ). Formic acid was purchased from Aldrich Chemical Co. (Milwaukee, WI). Heparin sodium and saline were obtained from Choong Wae Pharma (Seoul, Korea).

### Animal experiments

All animal care and the protocols for the fimasartan pharmacokinetic studies were conducted according to the Guidelines for the Care and Use of Animal which were approved by the Ethics Committee for Treatment of Laboratory Animal at Boryung Pharm. Co. Ltd.

Female SD rats (8–10 weeks old, body weight 250–330 g) were supplied from Orientbio Inc. (Daejeon, Korea) and kept in an animal facility with a 12 h light/dark cycle, a temperature of 23 ± 3 °C, relative humidity of 55 ± 15 %, and 10–20 air changes per hour. Polycarbonate cages with collection funnels (W 235 × L 380 × H 175 mm) were used to maintain 3 animals per cage during the inspection and acclimation period and 1 animal per cage during the dosing/observation period. All animals had free access to the pelleted rat diet which was sterilized by irradiation and provided by Samyang Co. (Seoul, Korea).

#### Maternal-fetal transfer of FMS

After 16–17 of gestation day (GD) when the fetal sizes are sufficient for analysis, female rats were anesthetized by intra-peritoneal injection of Zoletil 50 (20 mg/kg), and polyethylene tubing (Natume Co., Tokyo, Japan) was inserted to the jugular vein (SP45: 0.58 mm i.d., 0.96 mm o.d.) and femoral artery (SP28: 0.4 mm i.d., 0.8 mm o.d.). After 1 day of recovery, FMS dissolved in normal saline was administered via jugular vein by i.v. bolus dose of 2.70 and 5.50 mg/kg followed by constant i.v. infusion with rates of 0.17 and 0.34 mg/h/kg to achieve the target steady-state concentrations of 100 and 200 ng/mL, respectively. The initial i.v. bolus loading dose and the constant i.v. infusion rate were determined by multiplying the target steady-state concentration (C_ss_) to the steady-state volume of distribution (V_d,ss_ = 27.3 L/kg) and FMS clearance (28.6 mL/min/kg), respectively [13]. Doses were given in non-fasting conditions without anesthesia. Blood samples were collected in conscious animals at pre-dose, and 4, 8, 24, 28, and 32 h post-dose from the femoral artery. At each sampling time, approximately 0.3 mL of blood was collected and the same volume of heparinized saline (50 IU/mL) was added to the vein. Plasma samples were obtained by centrifugation of the blood samples at 13,000 × *g* for 5 min and stored at −20 °C until analysis. Three samples of each tissue, i.e., placenta, amniotic fluid, and the fetus were taken from one dam after sacrifice the dam by cervical dislocation under anesthesia (Zoletil 50, 2 mg/kg, i.v.) at 32 h after beginning the constant i.v. infusion. The placenta and fetus were homogenized by using a homogenizer (T10 basic, IKA, Wilmington, USA), after adding normal saline. Samples were stored at −20 °C until analysis. Steady-state plasma concentration was expressed as either the mean concentration of FMS at the 24–32 h period or the concentration at the last sampling time point (32 h). The average of the measured concentrations of each tissues taken from one dam was used to calculate the tissue to plasma partition coefficients (K_p_) by dividing the average tissue FMS concentration at 32 h by the steady-state plasma FMS concentration.

#### Mammary excretion of FMS

In mid-lactation period, on 12–13 lactation day (LD), female rats were anesthetized by intra-peritoneal injection of Zoletil 50 (20 mg/kg) and polyethylene tubing (Natume Co., Tokyo, Japan) was inserted to the jugular vein (SP45: 0.58 mm i.d., 0.96 mm o.d.) and femoral artery (SP28: 0.4 mm i.d., 0.8 mm o.d.). After 1 day of recovery, fimasartan dissolved in normal saline was administered via jugular vein by i.v. bolus dose of 2.70 and 5.50 mg/kg followed by constant i.v. infusion with rates of 0.17 and 0.34 mg/h/kg to achieve the target steady-state concentrations of 100 and 200 ng/mL, respectively. Doses were given in non-fasting conditions. Blood samples were collected at pre-dose, and 4, 8, 24, 28, and 32 h after post-dose. Milk was taken under mild anesthesia (Zoletil 50, 2 mg/kg, i.v.) at 32 h after starting constant i.v. infusion. Oxytocin 5 IU was injected subcutaneously at 30 min prior to the milk sampling in order to facilitate the collection of milk. Milk ejection was stimulated by gentle hand stripping of the teat, and the free milk flow was collected in polypropylene tubes. Samples were stored at −20 °C until analysis. Steady-state plasma concentration was expressed as either the mean concentration of FMS at the 24–32 h period or the concentration at the last sampling time point (32 h). The K_p_ for milk was calculated as the fraction of milk concentration over plasma FMS concentration at 32 h.

### Determination of FMS concentration by LC-MS/MS

The FMS concentrations in biological samples were determined by a modification of the previously reported LC-MS/MS assay [22]. Briefly, 200 μL of acetonitrile and 50 μL of the internal standard solution (BR-A-563 100 ng/mL in acetonitrile) were added to 50 μL of the thawed biological samples and mixed on a vortex mixer for 1 min. The sample mixture was then centrifuged for 10 min at 15,000 × g at 4 °C. The supernatant was transferred to a polypropylene tube and diluted with the same volume of distilled water. A volume of 10 μL was injected into LC-MS/MS.

The LC-MS/MS comprised API 2000 mass spectrometer (Applied Biosystems/MDS Sciex, Toronto, Canada) coupled with Waters 2690 HPLC system (Waters, Milford, MA). Fimasartan was separated on a Kinetex C_18_ column 50 × 2.10 mm, i.d., 2.6 μm (Phenomenex, Torrence, CA). The isocratic mobile phase composition was a mixture of acetonitrile and 0.05 % formic acid in water (40:60, v/v). The flow rate of the mobile phase was set at 0.2 mL/min, and the column oven temperature was 30 °C. The mass spectrometer was operated using electron spray ionization (ESI) with positive ion mode. The transition of the precursors to the product ion was monitored at 502.3→207.0 for fimasartan, and 526.4→207.2 for the internal standard (BR-A-563).

### Statistical analysis

The means of pharmacokinetic parameters were compared via unpaired *t*-tests for unpaired data. An ANOVA followed by Scheffe post hoc test was applied to compare means across three or more groups. *P* values <0.05 were considered as statistically significant. All the statistical analyses were conducted using SPSS (version 17.0, IBM Co., Armonk, NY, USA).

## Results

### Determination of FMS by LC-MS/MS

The lower limit of detection of the present assay was 0.5 ng/mL in the plasma, placenta, amniotic fluid, fetus, and milk matrices. The accuracy was 94.2–117.9 % in the plasma, 89.2–111.0 % in the placenta, 87.7–116.9 % in the amniotic fluid, 89.0–110.7 % in the fetus, and 88.8–109.5 % in the milk. The precisions were within 8.0, 12.3, 3.8, 10.4, and 8.5 % for plasma, placenta, amniotic fluid, fetus, and milk samples, respectively. The assay accuracy and precision data for matrix-matched quality control samples including lower limit of quantification are summarized in Table 1.

**Table 1:** Intra- and inter-day accuracy and precision of FMS assay for various matrices

Matrix			Concentration (ng/mL)			
			450	90	2	0.5
Plasma	Intra-day (*n* = 4)	Accuracy (%)	96.6	98.8	99.6	112.2
		Precision (CV %)	1.0	0.4	2.4	8.0
	Inter-day (*n* = 3)	Accuracy (%)	100.7	99.6	94.2	117.9
		Precision (CV %)	7.6	3.5	3.8	3.8
Placenta	Intra-day (*n* = 4)	Accuracy (%)	104.4	94.4	89.9	111.0
		Precision (CV %)	1.5	0.8	3.7	6.4
	Inter-day (*n* = 3)	Accuracy (%)	97.9	95.3	89.2	102.7
		Precision (CV %)	4.4	4.1	2.3	12.3
Amniotic Fluid	Intra-day (*n* = 4)	Accuracy (%)	102.3	100.0	97.0	114.8
		Precision (CV %)	0.4	0.7	2.9	2.5
	Inter-day (*n* = 3)	Accuracy (%)	99.6	95.4	87.7	116.9
		Precision (CV %)	2.1	3.8	2.0	1.2
Fetus	Intra-day (*n* = 4)	Accuracy (%)	98.7	96.1	89.0	98.5
		Precision (%)	1.4	1.4	2.3	10.1
	Inter-day (*n* = 3)	Accuracy (%)	94.9	93.0	97.0	110.7
		Precision (CV %)	0.9	0.7	7.8	10.4
Milk	Intra-day (*n* = 4)	Accuracy (%)	97.3	94.7	92.4	109.5
		Precision (CV %)	1.6	1.2	5.3	5.3
	Inter-day (*n* = 3)	Accuracy (%)	102.7	98.6	88.8	94.1
		Precision (CV %)	3.0	3.5	2.3	8.5

### Maternal-fetal transfer of FMS

**Fig. 1:**
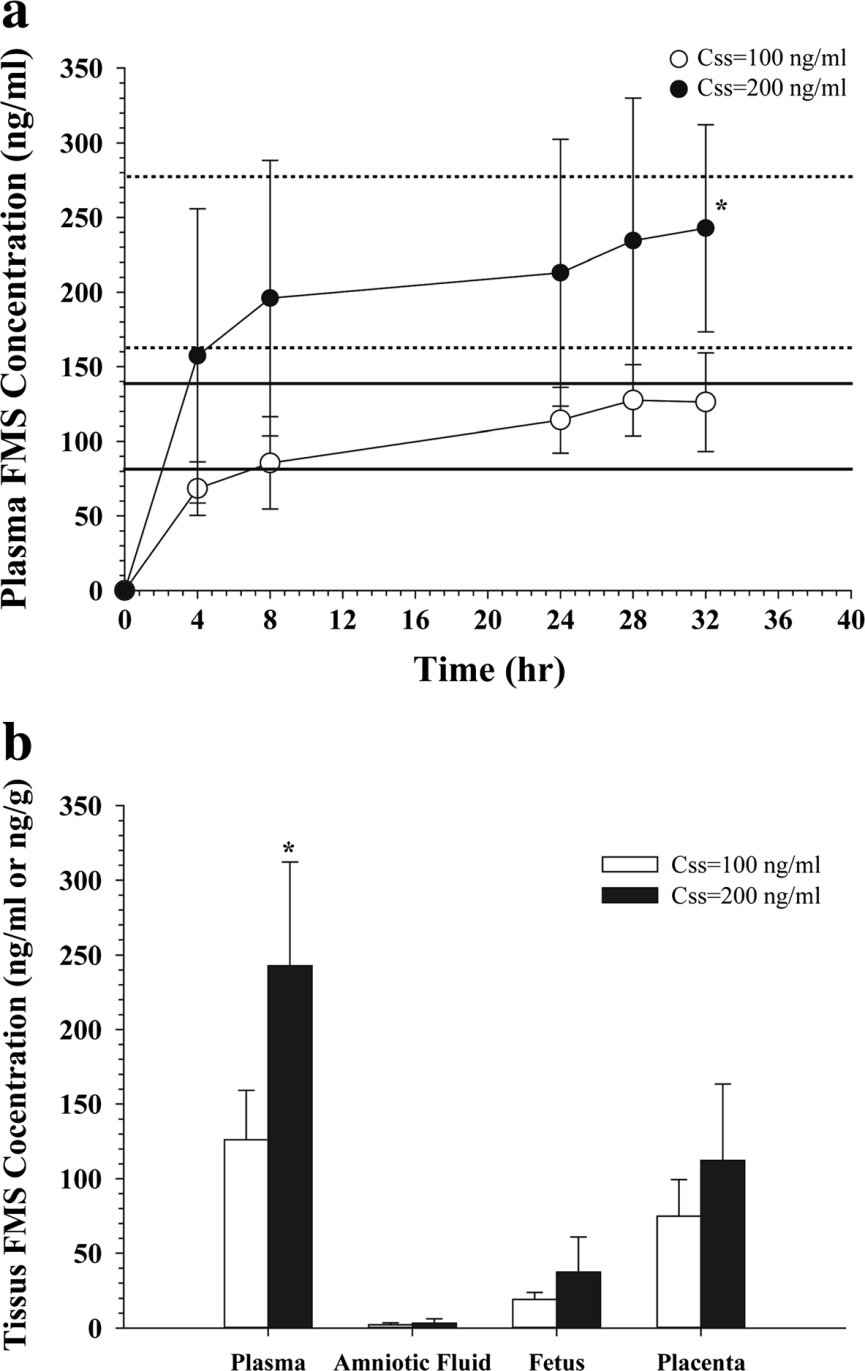
Fimasartan concentrations in **a** plasma and **b** tissues in pregnant rats after i.v. injection (2.7 and 5.5 mg/kg) followed by i.v. infusion (0.17 and 0.34 mg/h/kg) for target C_ss, plasma_ of 100 (*n* = 6) and 200 ng/mL (*n* = 5), respectively. *Bold* (target C_ss, plasma_ = 100 ng/mL) and *dashed* (target C_ss, plasma_ = 200 ng/mL) *lines* are the 10 and 90 % prediction intervals. Data are presented as the mean ± S.D. ^*^: *p* < 0.05, 100 vs. 200 ng/mL

The average plasma and tissue FMS concentration vs. time profiles following FMS administration to pregnant rats for the target steady-state concentrations (C_ss_) of 100 and 200 ng/mL are presented in Fig. 1. The prediction intervals of 10 and 90 % of C_ss_, calculated by FMS clearance derived from the previous rat i.v. bolus single dose study [13], are also presented (Fig. 1a). The calculated 10 and 90 % prediction intervals of C_ss_ were 81.4 and 138.6 ng/mL, respectively, for the target 100 ng/mL (when given constant i.v. infusion, rate = 0.17 mg/h/kg) and 162.8 and 277.3 ng/mL, respectively, for the target 200 ng/mL (when given constant i.v. infusion, rate = 0.34 mg/h/kg). The plasma FMS concentrations rapidly increased after FMS administration and reached 114. 1 ± 22.0 and 213.0 ± 89.4 ng/mL, which were close to the expected target C_ss_ = 100 and 200 ng/mL, respectively at 24 h. There was no significant difference among FMS concentrations at 24, 28, and 32 h post-dose, suggesting the plasma FMS have reached the steady state. FMS concentrations in the plasma as well as in the tissues, i.e., placenta, amniotic fluid, and fetus at 32 h post-dose were depicted in Fig. 1b. The C_ss_ = 200 ng/mL target dose group showed FMS concentrations of 112.2 ± 51.2 ng/g in the placenta while the C_ss_ = 100 ng/mL group showed 74.9 ± 24.5 ng/g. The FMS concentrations were 3.3 ± 3.0 vs. 2.3 ± 1.3 ng/mL in the amniotic fluid and 37.4 ± 23.6 vs. 19.2 ± 4.8 ng/g in the fetus in the C_ss_ = 200 ng/mL and C_ss_ = 100 ng/mL dose group, respectively. However, the differences in tissue concentrations between C_ss_ = 200 ng/mL vs. C_ss_ = 100 ng/mL groups were not statistically significant.

The average tissue-to-plasma partition coefficients (K_p_) are summarized in Table 2. While the placenta-to-plasma partition coefficient (K_p, placenta_) were 44.6–59.0 %, lower K_p_ values were observed for the amniotic fluid and fetus, which were 1.3–1.7 % and 14.9–17.0 %, respectively. The K_p_ values for all tissues were comparable between high (C_ss_ = 200 ng/mL) and low (C_ss_ = 100 ng/mL) dose groups, indicating that concentrations in the tested tissues proportionally increased as the plasma concentration increases and the fetal transfer of FMS was not affected by the dose.

**Table 2:** Average tissue-to-plasma partition coefficients in pregnant or lactating rats after i.v. injection (2.7 and 5.5 mg/kg) plus i.v. infusion (0.17 and 0.34 mg/h/kg) for 100 and 200 ng/mL of C_ss,plasma_, respectively

Tissue	Tissue to plasma partition coefficient, K_P_ (%)	
	100 ng/mL (*n* = 6)	200 ng/mL (*n* = 5)
Placenta	59.0 ± 7.9	44.6 ± 9.4
Amniotic fluid	1.7 ± 0.7	1.3 ± 0.9
Fetus	14.9 ± 1.3	17.0 ± 9.2
Milk	10.4 ± 6.0	15.2 ± 6.1

### Mammary excretion of FMS

**Fig. 2:**
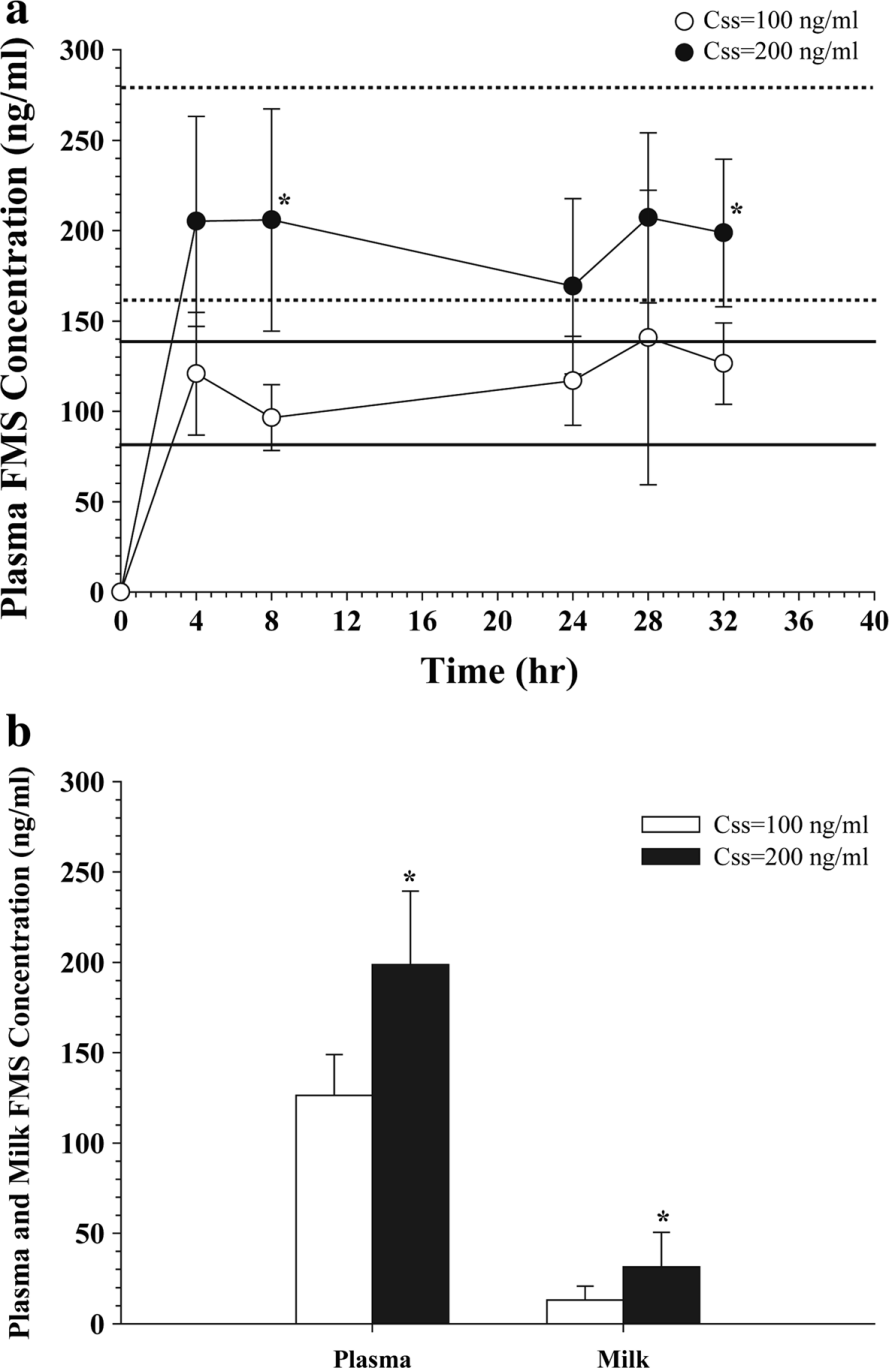
Fimasartan concentrations in **a** plasma and **b** milk in lactating rats after i.v. injection (2.7 and 5.5 mg/kg) followed by i.v. infusion (0.17 and 0.34 mg/h/kg) of fimasartan for target C_ss, plasma_ of 100 (*n* = 7) and 200 ng/mL (*n* = 5), respectively. *Bold* (target C_ss, plasma_ =100 ng/mL) and *dashed* (target C_ss, plasma_ =200 ng/mL) *lines* represent 10 and 90 % prediction intervals. Data are presented as the mean ± S.D. ^*^: *p* < 0.05, 100 vs. 200 ng/mL

The average FMS concentration in the plasma and milk vs. time profiles following FMS administration in rats 13 – 14 days after parturition for the target steady-state concentrations (C_ss_) of 100 and 200 ng/mL are presented in Fig. 2. The 10 and 90 % prediction intervals of C_ss_ for the target 100 ng/mL (81.4–138.6 ng/mL, following constant i.v. infusion at 0.17 mg/h/kg), and for the target 200 ng/mL (162.8–277.3 ng/mL, following constant i.v. infusion at 0.34 mg/h/kg) are also shown. The plasma FMS concentrations rapidly increased close to the target C_ss_ of 100 and 200 ng/mL and remained the target concentrations throughout the study period (Fig. 2a). There was no statistical difference in the plasma FMS concentrations among all the sampling times. At 32 h post-dose, plasma concentrations were 126.4 ± 49.3 and 198.8 ± 40.8 ng/mL in the target C_ss_ of 100 and 200 ng/mL groups, respectively. Plasma and milk FMS concentrations were significantly increased in a dose dependent manner. The C_ss_ = 200 ng/mL group resulted in significantly higher plasma and milk FMS concentrations than C_ss_ = 100 ng/mL group (*t*-test, *p* < 0.05) (Fig. 2b).

The calculated milk-to-plasma partition coefficients (K_p_) were 10.4–15.2 % (Table 2). Milk-to-plasma partition coefficients were comparable between dose groups (100 vs. 200 ng/mL) indicating that milk concentrations were proportionally increased as plasma concentration increases and the milk secretion was not affected by the dose.

## Discussion

Although reproductive toxicity studies suggested good safety profile of FMS during pregnancy and lactation in animals, the pharmacokinetic characteristics of FMS in disposition into fetus or breast milk are not known. The present study evaluated maternal-fetal transfer and mammary secretion of FMS in rats. The fetal disposition and secretion into milk was quantitatively determined at steady state when FMS was administered by constant intravenous infusion during pregnancy and lactation.

Our data suggest that the transfer of FMS to the amniotic fluid and fetus is relatively low with the tissue-to-plasma partition coefficients (K_p_) of 1.3–1.7 % for the amniotic fluid and 14.9–17.0 % for the fetus during late gestation (GD 17/18). The milk-to-plasma partition coefficient was 10.4–15.2 %. These fetal and mammary transfer of FMS are relatively lower compared to other ARBs. Placental transfer and mammary excretion of various ARBs are summarized in Table 3. Losartan was readily detected in the rat fetus, i.e., 26 % of the maternal exposure, following maternal administration during late gestation (GD 15–20) while fetal transfer was minimal in early gestation (GD 6–15) [17]. High fetal disposition of losartan was associated with the severe renal abnormalities in the F_1_ generation following oral administration to the mother during late gestation [23]. Substantial mammary secretion of losartan was also reported as more than 50 % of the plasma concentration was observed in the milk following repeated oral administration of losartan in LD 14 and 21 [17]. Significant fetal transfer of other ARBs was also reported. At last stage of gestation (GD 18), significant amount of telmisartan was distributed into fetus (Table 3). Following oral administration of telmisartan at a dose of 1 mg/kg, the fetal telmisartan concentration observed at terminal phase was two times higher than maternal plasma concentration. Milk excretion of telmisartan was also high as 150 % of plasma concentration was observed in milk at terminal phase [21]. Olmesartan [19] and valsartan [20] presented substantial fetal distribution with that more than 100 and 50 % of the maternal plasma concentration were observed, respectively.

**Table 3:** Summary of placental transfer and mammary excretion of various angiotensin receptor blockers

Disposition site	Drug	Dose (mg/kg)	Route	Gestation or Lactation day	Time after dose	Maternal Plasma conc. (PL)	Milk (MI) or Fetus (FE) conc.	K_P_ % (MI/PL or FE/PL)	Reference
Fetus	Losartan	135	P.O. (Multiple)	6 ~ 15	–	88^a^ (μg · hr/mL)	–	–	[17]
				15 ~ 20	–	194.8^a^ (μg · hr/mL)	50.7^a^ (μg · hr/mL)	26.0	
	Telmisartan	1	P.O.	12	4	43.56 (ng-eq/mL)	11.83 (ng-eq/g)	27.2	[18, 21]
				12	8	34.47 (ng-eq/mL)	11.03 (ng-eq/g)	32.0	
				12	24	5.98 (ng-eq/mL)	7.98 (ng-eq/g)	133.4	
				12	48	3.65 (ng-eq/mL)	1.89 (ng-eq/g)	51.8	
				18	4	77.05 (ng-eq/mL)	13.72 (ng-eq/g)	17.8	
				18	24	19.73 (ng-eq/mL)	45.27 (ng-eq/g)	229.4	
				18	48	9.01 (ng-eq/mL)	17.65 (ng-eq/g)	195.9	
	Olmesartan	–	P.O.	18	–	–	–	>100	[19]
	Valsartan	600	–	18	1	7 ~ 9 (μg-eq/g)	4 ~ 5 (μg-eq/g)	56.0	[20]
				18	24	ND^b^	3 ~ 4 (μg-eq/g)	–	
Milk	Losartan	135	P.O. (Multiple)	7	–	3.86 (μg/mL)	1.16 (μg/mL)	30.1	[17]
				14	–	3.00 (μg/mL)	1.96 (μg/mL)	65.3	
				21	–	3.14 (μg/mL)	1.71 (μg/mL)	54.5	
	Telmisartan	1	P.O.	–	0.5	23.77 (ng-eq/mL)	3.32 (ng-eq/mL)	14.0	[18, 21]
					2	31.04 (ng-eq/mL)	30.6 (ng-eq/mL)	98.6	
					4	34.84 (ng-eq/mL)	54.24 (ng-eq/mL)	155.7	
					8	36.12 (ng-eq/mL)	66.08 (ng-eq/mL)	182.9	
					24	9.3 (ng-eq/mL)	13.52 (ng-eq/mL)	145.4	
					48	4.06 (ng-eq/mL)	6.79 (ng-eq/mL)	167.2	
	Olmesartan	–	P.O.	–	72	–	–	25.0	[19]
	Valsartan	600	–	–	–	–	ND^b^	–	[20]

^a^Represented as AUC (μg · hr/mL), ^b^ *ND:* Not Detected

In most studies, fetal transfer and milk excretion of a drug have been determined following single administration, in which the K_p_ may be dependent on sampling time (Table 3). For example, K_p_ values of telmisartan in the fetus and milk were changed by the sampling time after single oral administration and it took more than several hours to achieve the steady state condition [21]. Even though the K_p_ values appeared to be stable after an equilibrium was achieved, this is a pseudo-equilibrium not a true equilibrium since equilibrium is continuously destroyed by elimination. The present study determined K_p_ of FMS in maternal-fetal units and milk at steady state following i.v. infusion. The K_p_ of FMS observed at steady state allowed us to evaluate the fetal transfer and mammary excretion more specifically without other confounding factors under true steady state condition.

Drug transfer into breast milk from maternal plasma is generally governed by passive diffusion. Factors such as ionization, plasma protein binding, molecular weight, lipophilicity, and the pharmacokinetics of a drug in the mother are known to determine mammary excretion [24, 25]. Involvement of carrier-mediated drug transport mechanisms in transfer into breast milk was also suggested [24]. In general, low plasma protein binding, low molecular weight, high lipophilic, and cationic drugs favor increased excretion into milk. The solubility of FMS is significantly changed depending on pH. FMS is an acidic drug with pKa 3.5 and solubility in water is increased from 0.017 to 4.34 mg/mL while logP is decreased from 1.76 to 0.37 as pH increased (6.0–8.0) (unpublished data). It is known that milk (pH = 7.2) is slightly more acidic than plasma (pH = 7.4), which may limit transfer of weakly acid drugs like FMS into breast milk by trapping them into plasma side by its ionization. This might be one of the potential reasons for limited mammary excretion of FMS.

The reasons for the different fetal disposition of FMS from other drugs in the class are not evident. One of the determinants of tissue distribution is the blood flow to the tissue. Since antihypertensive drugs alter the blood flow and different potency of the drugs may affect tissue distribution to different extent, the lower transfer of FMS may be associated with its higher potency compared to other ARBs. If the maternal-fetal transfer depends on the blood pressure, however, the transfer in higher dose group (C_ss_ = 200 ng/mL group) would have lower transfer (i.e., lower K_p_) than the lower dose group (C_ss_ = 100 ng/mL) in this study because the hypotensive effect of the higher dose is more significant. It has been reported that the in vivo antihypertensive effect of FMS is dose dependent at comparable plasma concentrations to the present study [13, 26]. Nevertheless, our data indicated that the K_p_fetus_ values were similar between different dose groups (Table 2). Thus, the lower fetal transfer of FMS is less likely due to a larger reduction in blood flow following decrease in arterial blood pressure compared to other ARBs. The transport of a drug across the placenta may occur by various mechanisms of ultrafiltration, simple and facilitated diffusion active transport, pinocytosis and breaks in placental villi, etc. [27]. As is simple diffusion usually the major mechanism, the lipid solubility of a drug is considered as a good index of its ability to cross the placenta, i.e., the more lipid soluble the drug the more readily it should cross the placenta. Nevertheless, it has been shown that lipophilicity cannot be the sole determinant of the transplacental transfer of several ACE inhibitors and ARBs [27]. In fact, the high lipophilicity of FMS is not consistent with the observed lower maternal-fetal transfer. Further studies are required to elucidate the mechanisms of the low fetal disposition of FMS.

The relatively low disposition of FMS into fetus or breast milk found in the present study may be associated with a good safety profile of FMS from the animal reproductive toxicity studies. However, the present finding may not be directly translated into toxicological beneficial effects on fetus compared to other ARBs due to the different receptor affinities and confounding factors in vivo. Whether the lower fetal and milk transfer of FMS is sufficient to lead to significant clinical benefit and safety during pregnancy and lactation needs to be evaluated.

## Conclusions

This study is the first report on the fetal and neonatal exposure to FMS. Our data indicated that FMS transfer to fetus and breast milk was relatively lower than other ARBs. Further studies are required to evaluate the clinical impact of the lower transfer of FMS into fetus and break milk as well as to uncover the potential mechanisms involved in FMS disposition into fetus or breast milk.

## Abbreviations

ACE, angiotensin-converting enzyme; ARB, angiotensin receptor blockers; C_ss_, steady-state concentration; FMS, fimasartan; GD, gestation day; K_p_, tissue to plasma partition coefficients; LC-MS/MS Liquid chromatography-tandem mass spectrometry; LD, lactation day; V_d,ss_, steady-state volume of distribution

## References

[1] National Heart L, Blood Institute. High blood pressure in pregnancy http://www.nhlbi.nih.gov/health/resources/heart/hbp-pregnancy. Accessed 30 March 2016.

[2] Podymow T, August P (2007). Hypertension in pregnancy. Adv Chronic Kidney Dis.

[3] Alwan S, Polifka JE, Friedman JM (2005). Angiotensin II receptor antagonist treatment during pregnancy. Birth Defects Res A Clin Mol Teratol.

[4] Chow KM, Lam CM (2004). Angiotensin II receptor antagonist in pregnancy. J Perinatol.

[5] Pietrement C, Malot L, Santerne B, Roussel B, Motte J, Morville P (2003). Neonatal acute renal failure secondary to maternal exposure to telmisartan, angiotensin II receptor antagonist. J Perinatol.

[6] Quan A (2006). Fetopathy associated with exposure to angiotensin converting enzyme inhibitors and angiotensin receptor antagonists. Early Hum Dev.

[7] Serreau R, Luton D, Macher MA, Delezoide AL, Garel C, Jacqz-Aigrain E (2005). Developmental toxicity of the angiotensin II type 1 receptor antagonists during human pregnancy: a report of 10 cases. BJOG.

[8] FDA US (2014). Safety: Cozaar (losartan potassium) 25 mg, 50 mg, and 100 mg Tablets.

[9] Mastrobattista JM (1997). Angiotensin converting enzyme inhibitors in pregnancy. Semin Perinatol.

[10] Spence SG, Cukierski MA, Manson JM, Robertson RT, Eydelloth RS (1995). Evaluation of the reproductive and developmental toxicity of the AT1-selective angiotensin II receptor antagonist losartan in rats. Teratology.

[11] Chung NA, Lip GY, Beevers M, Beevers DG (2001). Angiotensin-II-receptor inhibitors in pregnancy. Lancet.

[12] Martinovic J, Benachi A, Laurent N, Daikha-Dahmane F, Gubler MC (2001). Fetal toxic effects and angiotensin-II-receptor antagonists. Lancet.

[13] Kim TH, Shin S, Bashir M, Chi YH, Paik SH, Lee JH (2014). Pharmacokinetics and metabolite profiling of fimasartan, a novel antihypertensive agent, in rats. Xenobiotica.

[14] Chi YH, Lee H, Paik SH, Lee JH, Yoo BW, Kim JH (2011). Safety, tolerability, pharmacokinetics, and pharmacodynamics of fimasartan following single and repeated oral administration in the fasted and fed states in healthy subjects. Am J Cardiovasc Drugs.

[15] Lee HW, Lim MS, Seong SJ, Lee J, Park J, Seo JJ (2011). Effect of age on the pharmacokinetics of fimasartan (BR-A-657). Expert Opin Drug Metab Toxicol.

[16] Park JB, Sung KC, Kang SM, Cho EJ (2013). Safety and efficacy of fimasartan in patients with arterial hypertension (Safe-KanArb study): an open-label observational study. Am J Cardiovasc Drugs.

[17] Spence SG, Zacchei AG, Lee LL, Baldwin CL, Berna RA, Mattson BA (1996). Toxicokinetic analysis of losartan during gestation and lactation in the rat. Teratology.

[18] FDA US (1998). NDA #20-850.

[19] FDA US. NDA #21-286. 2002. http://www.accessdata.fda.gov/drugsatfda_docs/nda/2002/21-286_Benicar_pharmr_P6.pdf.

[20] Thomas M, Johnston C (2004). Valsartan. J Drug Eval.

[21] Wienen W, Entzeroth M, van Meel JCA, Stangier J, Busch U, Ebner T (2000). A review on telmisartan: a novel, long-acting angiotensin II-receptor antagonist. Cardiovasc Drug Rev.

[22] Shin BS, Kim TH, Paik SH, Chi YH, Lee JH, Tan HK (2011). Simultaneous determination of fimasartan, a novel antihypertensive agent, and its active metabolite in rat plasma by liquid chromatography-tandem mass spectrometry. Biomed Chromatogr.

[23] Spence SG, Allen HL, Cukierski MA, Manson JM, Robertson RT, Eydelloth RS (1995). Defining the susceptible period of developmental toxicity for the AT1-selective angiotensin II receptor antagonist losartan in rats. Teratology.

[24] Ito S, Lee A (2003). Drug excretion into breast milk—Overview. Adv Drug Deliv Rev.

[25] Atkinson UC, Begg E (1990). Prediction of drug distribution into human milk from physicochemical characteristics. Clin Pharmacokinet.

[26] Chi YH, Lee JH, Kim JH, Tan HK, Kim SL, Lee JY (2013). Pharmacological characterization of BR-A-657, a highly potent nonpeptide angiotensin II receptor antagonist. Biol Pharm Bull.

[27] Stevenson KM, Gibson KJ, Lumbers ER (1995). Comparison of the transplacental transfer of enalapril, captopril and losartan in sheep. Br J Pharmacol.

